# FGF22 promotes generation of ribbon synapses through downregulating MEF2D

**DOI:** 10.18632/aging.103042

**Published:** 2020-04-09

**Authors:** Shuna Li, Jingchun He, Yupeng Liu, Jun Yang

**Affiliations:** 1 Department of Otolaryngology-Head and Neck Surgery, Xinhua Hospital, Shanghai Jiaotong University School of Medicine, Shanghai 200092, China

**Keywords:** hearing loss, ribbon synapse, inner hair cells, FGF22, MEF2D

## Abstract

Cochlear ribbon synapses play a pivotal role in the prompt and precise acoustic signal transmission from inner hair cells (IHCs) to the spiral ganglion neurons, while noise and aging can damage ribbon synapses, resulting in sensorineural hearing loss. Recently, we described reduced fibroblast growth factor 22 (FGF22) and augmented myocyte enhancer factor 2D (MEF2D) in an ototoxicity mouse model with impaired ribbon synapses. Here, we investigated the mechanisms that underlie the FGF22/MEF2D- regulated impairment of ribbon synapses. We generated adeno-associated virus (AAV) carrying FGF22, shFGF22, MEF2D, shMEF2D, calcineurin (CalN), shCalN or corresponding scramble controls for transduction of cultured mouse hair cells. We found that FGF22 was a suppressor for MEF2D, but not vice versa. Moreover, FGF22 likely induced increases in the calcium influx into IHCs to activate CalN, which subsequently inhibited MEF2D. Cochlear infusion of AAV-shFGF22 activated MEF2D, reduced ribbon synapse number and impaired hearing function, which were all abolished by co-infusion of AAV-shMEF2D. Hence, our data suggest that the ribbon synapses may be regulated by FGF22/calcium/CalN/MEF2D signaling, which implied novel therapeutic targets for hearing loss.

## INTRODUCTION

Hearing relies on faithful synaptic transmission at the ribbon synapse of auditory hair cells [1]. One row of inner hair cells (IHCs) and three rows of outer hair cells (OHCs) constitute human cochlear hair cells, while the IHCs are the actual sensory receptors, which connect with 95% of the fibers of the auditory nerve projected to the brain [2]. Healthy cochlear ribbon synapses are critical for prompt and precise transmission of acoustic signals including frequency, intensity and timing information from IHCs to the spiral ganglion neurons [3].

Hearing loss can result from both cochlear hair cell death and ribbon synapse damage [4]. Indeed, acoustic overexposure, as well as aging, could damage ribbon synapses to cause sensorineural hearing loss [4]. The damage of ribbon synapses appeared earlier than apoptosis of IHCs, which do not regenerate throughout adult life [2]. Moreover, IHC ribbon synapses are susceptible to ototoxicity, and they respond by altering ribbon synapse number [5].

Fibroblast growth factor 22 (FGF22) is a member of FGF7 subfamily and signals through its binding to FGFR2b [6]. Previous studies have demonstrated that FGF22 is specifically functional in neural system. For example, FGF22 was found to mediate synaptogenesis in the adult nervous system to control the synapse regeneration and maturation during post-injury repair in the spinal cord [7]. FGF22 is specifically expressed in HCs [8], and is required for the induction of excitatory synapses [9], and for glutamatergic presynaptic differentiation [10]. Moreover, the synapse size in CA3 region of mouse brain has been found regulated by FGF22 [11]. Recently, we described that reduced FGF22 and augmented myocyte enhancer factor 2D (MEF2D) appear to be responsible for the impaired ribbon synapses by ototoxic agents [12]. All these studies suggest a potential role of FGF22 in the regulation of ribbon synapses. However, the related biological molecular mechanisms are still unclear.

Here, we investigated the mechanisms that underlie the FGF22/MEF2D- regulated impairment of ribbon synapses. We generated adeno-associated virus (AAV) carrying FGF22, shFGF22, MEF2D, shMEF2D, calcineurin (CalN), shCalN or corresponding scramble controls for transduction of cultured mouse hair cells. We found that FGF22 was a suppressor for MEF2D, but not vice versa. Moreover, FGF22 likely induced increases in the calcium influx into IHCs to activate CalN, which subsequently inhibited MEF2D. Cochlear infusion of AAV-shFGF22 activated MEF2D, reduced ribbon synapse number and impaired hearing function, which were all abolished by co-infusion of AAV-shMEF2D.

## RESULTS

### Modulation of FGF22 and MEF2D levels in the cultured mouse hair cells

**Figure 1:**
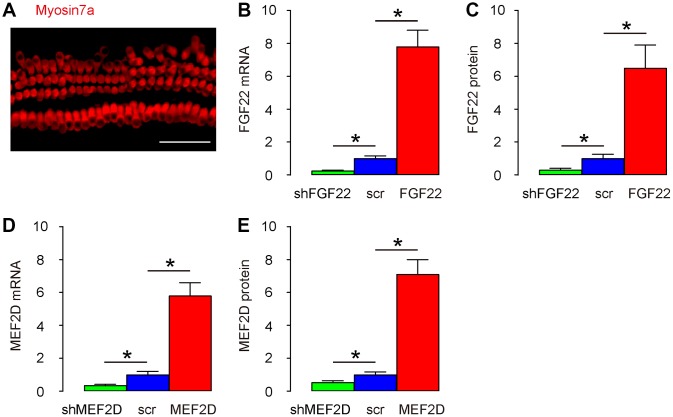
**Modulation of FGF22 and MEF2D levels in the cultured mouse hair cells.** (**A**) Isolated mouse hair cells in culture and immunostained with Myosin7a (in red). (**B**, **C**) Cultured hair cells were transduced with AAV-FGF22 or AAV-shFGF22 or control AAV-scr (scramble) and assessed for FGF22 levels by RT-qPCR (**B**), and by ELISA (**C**). (**D**, **E**) Cultured hair cells were transduced with AAV-MEF2D or AAV-shMEF2D or control AAV-scr (scramble) and assessed for MEF2D levels by RT-qPCR (**D**), and by ELISA (**E**). *p<0.05. N=5. Scale bar is 50μm.

Mouse hair cells were isolated and kept in culture and their structure was shown after immunostaining for Myosin7a, a specific marker for hair cells, in a representative image (Figure 1A). In order to study the mechanisms by which the FGF22/MEF2D regulates the alteration in ribbon synapses, we generated AAV vectors overexpressing or depleting FGF22 and MEF2D, respectively. First, these viral vectors were validated by transducing cultured hair cells. We found that AAV-FGF22 significantly increased mRNA of FGF22 in hair cells, while AAV-shFGF22 significantly decreased mRNA of FGF22 in hair cells, by RT-qPCR (Figure 1B). Moreover, AAV-FGF22 significantly increased protein of FGF22 in hair cells, while AAV-shFGF22 significantly decreased protein of FGF22 in hair cells, by ELISA (Figure 1C). We also found that AAV-MEF2D significantly increased mRNA of MEF2D in hair cells, while AAV-shMEF2D significantly decreased mRNA of MEF2D in hair cells, by RT-qPCR (Figure 1D). AAV-MEF2D significantly increased protein of MEF2D in hair cells, while AAV-shMEF2D significantly decreased protein of MEF2D in hair cells, by ELISA (Figure 1E). Thus, these FGF22/MEF2D-modulating AAVs faithfully altered the FGF22/MEF2D levels in mouse hair cells.

### FGF22 suppresses MEF2D in the cultured mouse hair cells

**Figure 2:**
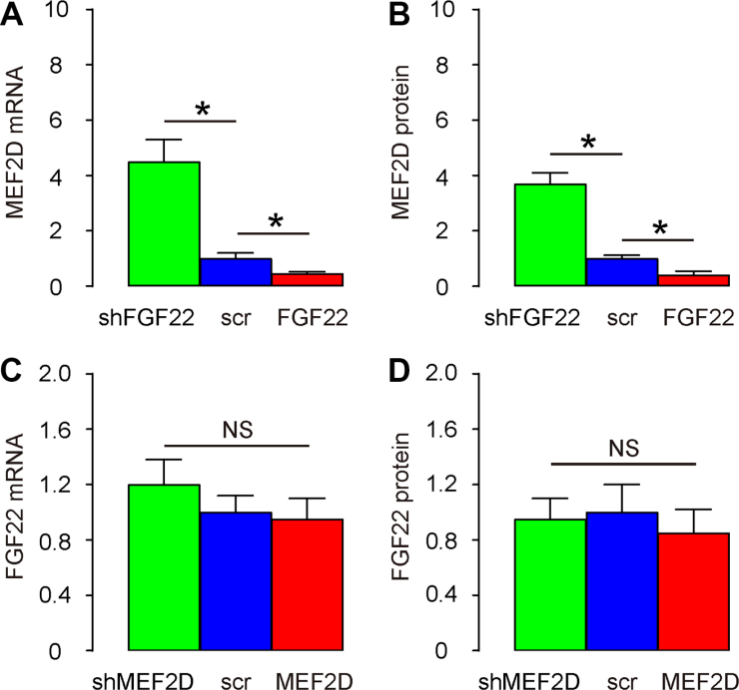
**FGF22 suppresses MEF2D in the cultured mouse hair cells.** (**A**, **B**) Cultured hair cells were transduced with AAV-FGF22 or AAV-shFGF22 or control AAV-scr (scramble) and assessed for MEF2D levels by RT-qPCR (**A**), and by ELISA (**B**). (**C**, **D**) Cultured hair cells were transduced with AAV-MEF2D or AAV-shMEF2D or control AAV-scr (scramble) and assessed for FGF22 levels by RT-qPCR (**C**), and by ELISA (**D**). *p<0.05. N=5.

Next, we examined the regulatory relationship between FGF22 and MEF2D. We found that AAV-FGF22 significantly decreased mRNA of MEF2D in hair cells, while AAV-shFGF22 significantly increased mRNA of MEF2D in hair cells, by RT-qPCR (Figure 2A). Moreover, AAV-FGF22 significantly decreased protein of MEF2D in hair cells, while AAV-shFGF22 significantly increased protein of MEF2D in hair cells, by ELISA (Figure 2B). Thus, FGF22 suppresses MEF2D in the cultured mouse hair cells. On the other hand, neither AAV-MEF2D nor AAV-shMEF2D significantly altered mRNA (Figure 2C) or protein (Figure 2D) of FGF22 in hair cells, suggesting that MEF2D does not regulate FGF22 in the cultured mouse hair cells. Hence, FGF22 is the upstream regulator for MEF2D, but not vice versa.

### FGF22 increases calcium influx in the cultured mouse hair cells

**Figure 3:**
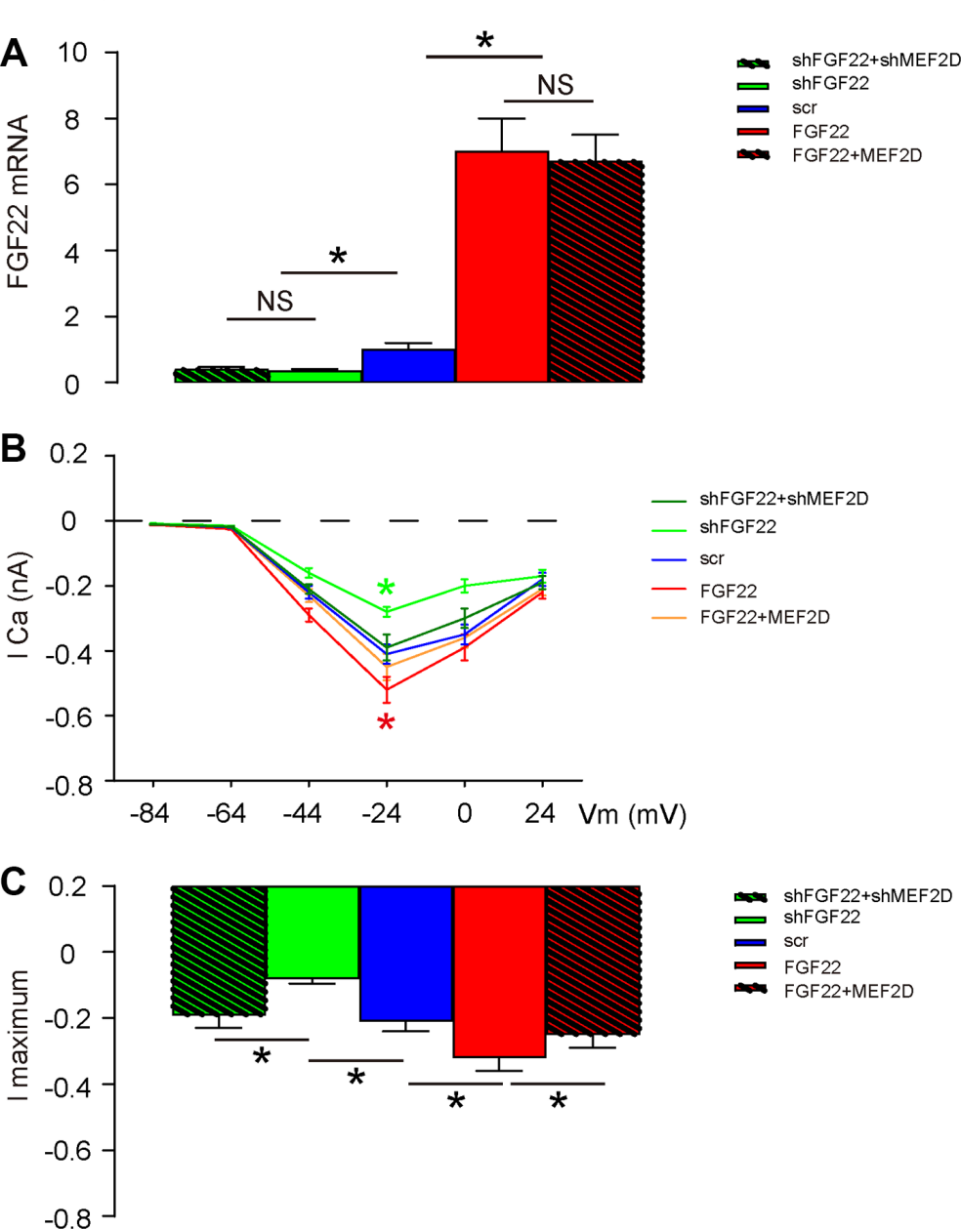
**FGF22 increases calcium influx in the cultured mouse hair cells.** The hair cell voltage-gated Calcium current was measured in AAV-FGF22- or AAV-shFGF22-transduced hair cells. AAV-MEF2D was also co-transduced hair cells with AAV-FGF22 and AAV-shMEF2D was also co-transduced hair cells with AAV-shFGF22, both aiming to abolish the effects of FGF22 modulation on MEF2D. (**A**) RT-qPCR for FGF22 mRNA levels by co-transduction with different AAVs. (**B**, **C**) Measurement of current-voltage in transduced cells, by lCa (**B**), and by l maximum (**C**). *p<0.05. NS: non-significant. N=5.

Previous studies have shown that FGF22 regulates calcium currency in cells to drive exocytosis [13, 14], and calcium has been shown as a suppressor for MEF2 [15]. Thus, we hypothesized that FGF22 may inhibit MEF2D through increases in calcium influx. To prove it, we measured the hair cell voltage-gated calcium current in AAV-FGF22- or AAV-shFGF22-transduced hair cells. AAV-MEF2D was also co-transduced with AAV-FGF22, while AAV-shMEF2D was also co-transduced with AAV-shFGF22, aiming to abolish the effects of FGF22 modulation on MEF2D. First, we confirmed that alteration of FGF22 levels by FGF22-AAVs was not affected by co-transduction with MEF2D-AAVs (Figure 3A). Next, we measured current-voltage in these transduced cells. We found that overexpression of FGF22 significantly increased calcium currency in cells, which was completely abolished by co-expression of MEF2D, while of depletion of FGF22 significantly decreased calcium currency in cells, which was completely abolished by co-expression of shMEF2D (Figure 3B–3C). These data suggest that FGF22 indeed increases calcium influx in the cultured mouse hair cells.

### FGF22-induced calcium influx activates CalN to inhibit MEF2D in mouse hair cells

**Figure 4:**
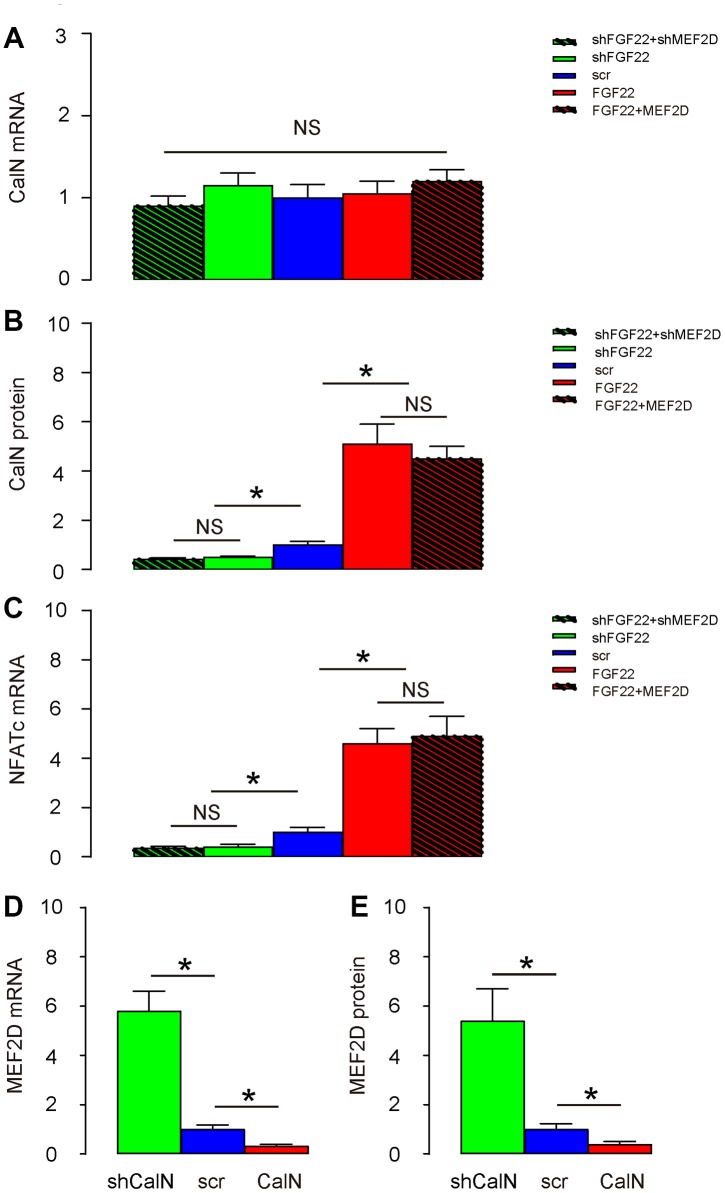
**FGF22-induced calcium influx activates CalN to inhibit MEF2D in mouse hair cells.** (**A**, **B**) CalN levels in transduced hair cells by RT-qPCR (**A**), and by ELISA (**B**). (**C**) NFATc levels in transduced hair cells by RT-qPCR. (**D**, **E**) Cultured hair cells were transduced with AAV-CalN or AAV-shCalN or control AAV-scr (scramble) and assessed for MEF2D levels by RT-qPCR (**D**), and by ELISA (**E**). *p<0.05. NS: non-significant. N=5.

CalN is a calcium-dependent serine/threonine protein phosphatase. Increased calcium influx activates CalN by binding a regulatory subunit of nuclear factor of activated T cell cytoplasmic (NFATc), and then CalN induces transcription factors (NFATc) to mediate multiple functions, including suppression of MEF2 [16, 17]. Thus, we examined the expression of CalN in these conditions. While CalN mRNA remained unaltered in hair cells transduced with these 5 different combinations of AAVs (Figure 4A), we found that overexpression of FGF22 significantly increased protein levels of CalN and its downstream factor NFATs in cells, both of which were completely abolished by co-expression of MEF2D, while of depletion of FGF22 significantly decreased protein levels of CalN and its downstream factor NFATs in cells, both of which were completely abolished by co-expression of shMEF2D (Figure 4B, 4C), suggesting that FGF22-induced calcium influx activates CalN in mouse hair cells. In order to find out whether CalN by itself regulates MEF2D, we generated CalN-modulating AAVs to transduce hair cells. We found that AAV-CalN significantly decreased mRNA of MEF2D in hair cells, while AAV-shCalN significantly increased mRNA of MEF2D in hair cells, by RT-qPCR (Figure 4D). Moreover, AAV-CalN significantly decreased protein of MEF2D in hair cells, while AAV-shCalN significantly increased protein of MEF2D in hair cells, by ELISA (Figure 4E). Together, these data suggest that FGF22-induced calcium influx activates CalN to inhibit MEF2D in mouse hair cells.

### FGF22 depletion reduces ribbon synapse number in vivo

**Figure 5:**
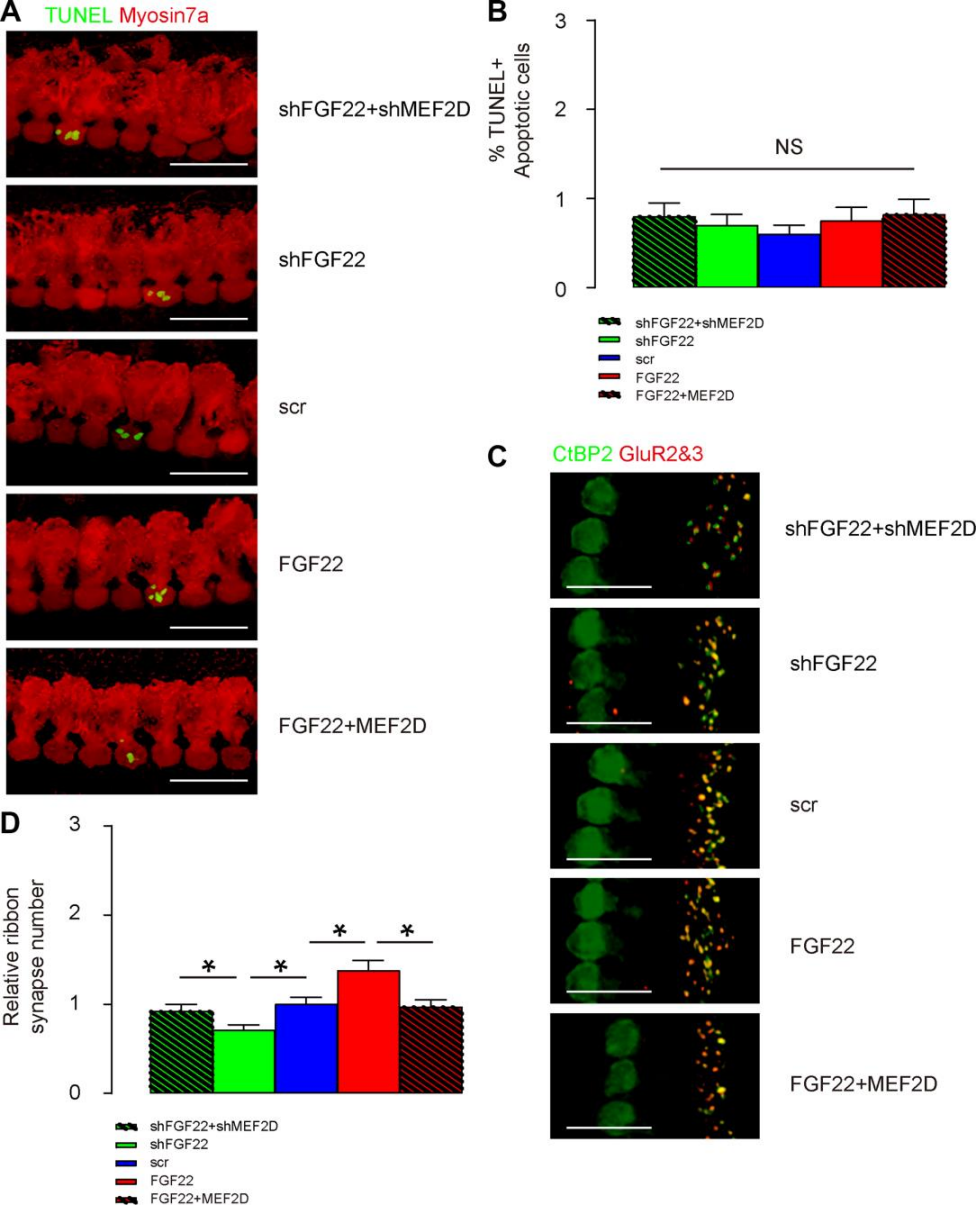
**FGF22 depletion reduces ribbon synapse number in vivo.** AAV-shFGF22 and AAV-FGF22 were administrated to mouse cochlea to deplete or overexpress FGF22 in hair cells, respectively. Co-administration of AAV-shMEF2D with AAV-shFGF22 was also performed to assess the regulatory relationship between FGF22 and MEF2D in hair cells and ribbon synapses. Similarly, co-administration of AAV-MEF2D with AAV-FGF22 was performed, also to assess the regulatory relationship between FGF22 and MEF2D in hair cells and ribbon synapses. (**A**, **B**) TUNEL staining was performed on cochlea, showing by representative images (**A**), and by quantification (**B**). (**C**, **D**) The ribbon synapses were determined by co-staining for CtBP2 (in green) and GluR2&3 (in red). Cochlear ribbon synapse number was assessed, shown by representative images (**C**), and by quantification (**D**). *p<0.05. NS: non-significant. N=5. Scale bar is 20μm.

AAV-shFGF22 and AAV-FGF22 were administrated to mouse cochlea to deplete or overexpress FGF22 in hair cells, respectively. Co-administration of AAV-shMEF2D with AAV-shFGF22 or co-administration of AAV-MEF2D with AAV-FGF22 was also performed to assess the regulatory relationship between FGF22 and MEF2D in hair cells and ribbon synapses. First, TUNEL staining was performed on cochlea, showing no alteration in cell apoptosis by either AAV combinations, shown by representative images (Figure 5A), and by quantification (Figure 5B). Thus, FGF22/MEF2D does not regulate hair cell apoptosis. The ribbon synapses were determined by immunopositivity for presynaptic membrane marker CtBP2 and postsynaptic membrane marker GluR2&3. We found that overexpression of FGF22 significantly increased the ribbon synapse number in cochlea, which was completely abolished by co-expression of MEF2D, while of depletion of FGF22 significantly decreased ribbon synapse number in cochlea, which was completely abolished by co-expression of shMEF2D, shown by representative images (Figure 5C), and by quantification (Figure 5D). These data suggest that FGF22 depletion reduces ribbon synapse number in vivo.

### FGF22 depletion induces elevation of ABR threshold

**Figure 6:**
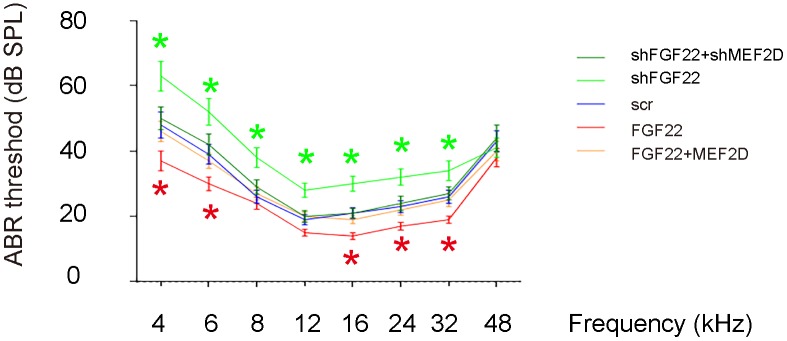
**FGF22 depletion induces elevation of ABR threshold.** The effects on ABR threshold of the mice were assessed. ABR threshold was identified as the stimulus intensity required to evoke a voltage response 5x the root mean square (RMS) noise floor for the measurement. *p<0.05. N=5.

**Figure 7:**
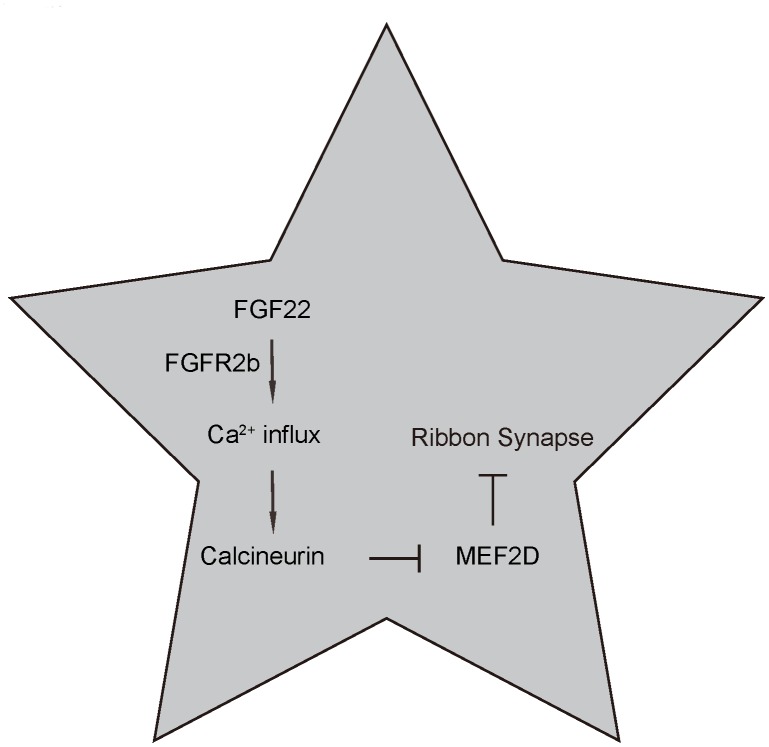
**Schematic of the model.** Regulation of ribbon synapses by FGF22/calcium/CalN/MEF2D signaling. FGF22 signaling through its major receptor FGFE2b triggers an increase of intracellular Ca^2+^, which activates the CaN and its downstream factors, e.g. NFATc. Activated CaN subsequently suppresses MEF2D, the inhibitory effects of which on ribbon synapses are then released.

Next, we assessed the effects on ABR threshold of the mice. ABR threshold was identified as the stimulus intensity required to evoke a voltage response 5x the root mean square (RMS) noise floor for the measurement. We found that overexpression of FGF22 significantly reduced the ABR threshold, which was completely abolished by co-expression of MEF2D, while of depletion of FGF22 significantly increased the ABR threshold, which was completely abolished by co-expression of shMEF2D (Figure 6). These data suggest that FGF22 depletion induces elevation of ABR threshold. Our study was thus summarized in a schematic (Figure 7).

## DISCUSSION

Unlike synapses in the central nervous system, cochlear ribbon synapses are the first synapses in the process of transmitting sound to the central nerve system [2]. Auditory formation relies on quick and precise release of intra-tactile neurotransmitters, which determines the number and quality of acoustic signals passed to the central system. We have previously shown that intraperitoneal injection of gentamycin-induced ototoxicity and hearing impairment through disruption of ribbon synapses without immediate effects on the apoptotic death of IHCs and OHCs [12]. Moreover, reduction in the number of ribbon synapses occurred concomitantly with decrease in FGF22 and increase in MEF2D [12]. Furthermore, gentamycin-induced ototoxicity and hearing impairment were attenuated by FGF22 infusion into the mouse cochlea [12].

In the current study, we used a set of gain-of-function or loss-of-function experiments, to further demonstrate the regulatory axis of FGF22 and MEF2D in the integrity and mass maintenance of ribbon synapses. First, we showed the regulatory direction is from FGF22 to MEF2D but not vice vista. Hence, the MEF2D is downstream of FGF22. Of note, MEF2 reduction in hippocampal neurons was found to be associated with increases in number of glutamatergic synapses, and overexpression of MEF2 reduced excitatory synapse number [18]. In granule neurons of the cerebellum, MEF2 suppressed differentiation of postsynaptic dendritic claws [19]. To the best of our knowledge, our studies are the first to demonstrate a role of MEF2D in the control of ribbon synapse number. Since the ability of MEF2 to regulate synapse number in other neuronal types is dependent on calcium signaling pathways [18, 19], we performed analysis on calcium influx and CalN. We found that CalN was not only the target factor to be activated by FGF22-mediated calcium influx, but also the suppressor of MEF2D expression. Our data showed that the activation of CalN by FGF22-mediated calcium influx was not at transcription level, which was consistent with the role of calcium as a co-factor for activation and function of CalN [13, 14]. On the other hand, CalN inhibited MER2D at both transcriptional and translational levels, which was consistent with previous reports [16, 17].

In addition, FGF has been found to activate CalN to antagonize the BMP signaling pathway for neural induction [20], and MEF2 could be activated by BMP4 [21]. Thus, FGF22 might contribute to preservation of IHC ribbon synapse and hearing function through modulating BMP signaling. This possible mechanism, in addition to the determined FGF22/calcium/CalN/MEF2D signaling, may be also responsible for the observation in the current study.

To summarize, here we reported a novel molecular signaling pathway that governs the integrity and function of ribbon synapses during hearing transmission, which may imply novel therapeutic targets to antagonize hearing loss.

## MATERIALS AND METHODS

### Animal and experimental protocols

All experimental protocols were approved by the Animal Care and Use Committee of Shanghai Jiaotong University. All experiments were performed in accordance with the guidelines from the Animal Research and Care Committee at Shanghai Jiaotong University. Specific pathogen free (SPF) male CBA/J mice (aged 6 weeks, weight around 18g) were supplied by Shanghai Model Organisms Center (Shanghai, China). Mice with outer or middle ear disease were excluded from this study. Mice were anesthetized with isoflurane inhalation followed by intraperitoneal injection of ketamine (150 mg/kg), xylazine (6 mg/kg) for physiological tests.

### AAV vectors

Small hairpin interfering RNAs (shRNA) against FGF22 (shFGF22), FGF22, shRNA against MEF2D (shMEF2D), MEF2D, shRNA against CalN (shCalN), CalN and respective scramble sequences were inserted into a backbone pcDNA3.1-CMV-GFP plasmid (Clontech, Mountain View, CA, USA). AAV vectors were generated by co-transfection of the expression plasmid, a pHelper plasmid, and a serotype 2 rep-cap vector into human embryonic kidney 293 cells with Lipofectamine 3000 reagent (Invitrogen, Shanghai, China), according to the instructions of the manufacturer. Titration of viral vectors was determined using a dot-blot assay. The prepared virus was stored at -80°C.

### Hair cell isolation and culture

The cochlea was collected from 6-week-old CBA/J mice. The mouse cochleae were removed from temporal bone, and further dissected in cold 0.01 mM PBS under a dissecting microscope, by which the inner and outer hair cells and support cells were separated from the large epithelium in Hank's solution to obtain a complete sensory epithelium without spiral neurons. Mouse hair cells were maintained in a defined medium in culture: DMEM-F12 (Thermo Scientific, Rockford, IL, USA) with B27 supplement (Thermo Scientific), 1 mM n-acetyl-l-cysteine (Sigma-Aldrich, St. Louis, MO, USA), penicillin–streptomycin (Thermo Scientific), and 20 ng/ml epidermal growth factor (Sigma-Aldrich), in a 5% CO2/5% O2 humidified incubator. Cultures were fed weekly.

### AAV infusion

Alzet mini-osmotic pumps with catheters (Model 1004, reservoir volume 100 mL; Alzet, CA, USA) were used to infuse AAVs (10^10^ in 100ml) solution into the cochlea at the flow rate of 0.11 mL/h for 4 days, as described before [12]. Briefly, after anesthesia, a postauricular incision of left ear was generated to open the bulla to identify round window (RW) niche, after which the catheter tip was inserted into a drilled hole close to the round window in the basal turn of the cochlea. The bulla was closed with dental cement and the osmotic pump was fixed in a superficial subcutaneous pocket in the back of the mice.

### ABR auditory measurements

The auditory brainstem responses (ABRs) of the mice were assessed as previously described [12]. Mice were anesthetized by administering 150 mg/kg ketamine and 6 mg/kg xylazine intraperitoneally and their body temperature was held constantly at 37°C with FHC, DC-temperature controller and heating pad until their full recovery. ABRs were recorded by placing three needle electrodes subcutaneously at the vertex, below the left ear, and a ground electrode close to the tail. The signals were amplified 10,000 times using a biological amplifier (Warner Instruments, DP-311) digitized at 10 kHz, and digitally band-pass filtered from 300 to 3,000 Hz. At each frequency, the peak to peak voltages of ABR signals, at stimulus intensities ranging from 10 to 80 dB sound pressure levels (SPL), were measured in 10 dB steps and fitted and interpolated to find thresholds five standard deviations above the noise floor. ABR data were acquired using a computer-based signal-averaging system from SmartEP (Intelligent Hearing Systems, USA). Hearing threshold was determined by ABR waveforms and was defined as the lowest distinguishable stimulus sound pressure.

### Immunostaining and ELISA

Cochlear tissue from mouse or culture was assessed for protein expression by immunohistochemistry or by ELISA. For immunohistochemistry, the tissue was fixed in a 4% paraformaldehyde for 2 hours, then paraffin-embedded followed by being cut into 5-μm-thick sections. The immunohistochemistry used fluorescence-based method. Primary antibodies are rabbit anti-CtBP2 polyclonal antibody (ab128871, 1:100, Abcam, Cambridge, MA, USA), rabbit anti-GluR2&3 polyclonal antibody (2mg/mL, Fisher Scientific, CA, USA) and rabbit anti-Myosin7a (1:200, ab3481, Abcam). Secondary antibodies are cy2- or cy3- conjugated anti-rabbit (Jackson ImmunoResearch Labs, West Grove, PA, USA). TUNEL staining was performed with In Situ Cell Death Detection Kit (Roche, Indianapolis, IN, USA). ELISA was performed using mouse FGF22 ELISA kit (MBS2089564, MyBiosource Inc., San Diego, CA, USA), human/mouse MEF2D ELISA kit (LS-F913, LS-Bio, Seattle WA, USA), mouse Calcineurin ELISA kit (MBS730033, MyBiosource Inc.) and mouse NFATc ELISA kit (ab207215, Abcam), as instructed.

### Quantitative real-time PCR

The extraction of total RNA was performed with a high-purity total RNA extraction kit (BioTeke Ltd., Beijing, China). Total RNA was reverse transcribed using a Reverse Transcription kit (Qiagen GmbH, Hilden, Germany), according to the manufacturer’s protocol. The cDNA was then used for the detection of gene expression using a SYBR Green PCR kit (Qiagen GmbH). The primers used are FGF22: forward: 5’-GAGGCCCTGGCTGAGTAA-3’, reverse: 5’-ACACGGACAGAACGGATCTC-3’; MEF2D: forward: 5’-CGTTGGGAATGGCTATGTC-3’, reverse: 5’-GAGGCCCTGGCTGAGTAA-3’; CalN: forward: 5’-TGCAAAGCGCTACTGTTGAG-3’, reverse: 5’-GAGGTGGCATCCTCTCGTTA-3’; beta-actin: forward: 5’-AAGGACTCCTATAGTGGGTGACGA-3’; reverse: 5’-ATCTTCTCCATGTCGTCCCAGTTG-3’. The gene expression was normalized to beta-actin. Relative gene expression was calculated using the 2^–ΔΔCq^ method.

### Statistical analysis

All statistical analyses were carried out using the SPSS 20.0 statistical software package. Data were investigated using one-way ANOVA with a Bonferroni correction, followed by Fisher's exact test to compare 2 sub-groups. All values are shown as mean ± standard deviation (SD) and are considered significant if p < 0.05, not significant (NS) if p>0.05.
